# Regulation of plasma volume in male lowlanders during 4 days of exposure to hypobaric hypoxia equivalent to 3500 m altitude

**DOI:** 10.1113/JP280601

**Published:** 2020-11-16

**Authors:** Maja Schlittler, Hannes Gatterer, Rachel Turner, Ivo B. Regli, Simon Woyke, Giacomo Strapazzon, Peter Rasmussen, Michael Kob, Thomas Mueller, Jens P. Goetze, Marc Maillard, Gerrit van Hall, Eric Feraille, Christoph Siebenmann

**Affiliations:** ^1^ Institute of Mountain Emergency Medicine EURAC Research Bolzano Italy; ^2^ Department of Anesthesia and Intensive Care Medicine ‘F. Tappeiner’ Hospital Merano Italy; ^3^ Department of Anaesthesiology and Intensive Care Medicine Medical University of Innsbruck Austria; ^4^ H. Lundbeck A/S Valby Denmark; ^5^ Division of Clinical Nutrition Bolzano Regional Hospital Bolzano Italy; ^6^ Department of Clinical Pathology Hospital of Bolzano Bolzano Italy; ^7^ Department of Clinical Biochemistry Rigshospitalet University of Copenhagen Copenhagen Denmark; ^8^ Service of Nephrology University Hospital of Lausanne Lausanne Switzerland; ^9^ Department of Biomedical Sciences Faculty of Health and Medical Sciences University of Copenhagen Copenhagen Denmark; ^10^ Clinical Metabolomics Core Facility Rigshospitalet University of Copenhagen Denmark; ^11^ National Center of Competence in Research Kidney Control of Homeostasis (Kidney.CH) Zurich Switzerland; ^12^ Department of Cellular Physiology and Metabolism University of Geneva University Medical Center Geneva Switzerland

**Keywords:** diuresis, fluid balance, high altitude, hormones, total body water

## Abstract

**Key points:**

- Acclimatization to hypoxia leads to a reduction in plasma volume (PV) that restores arterial O_2_ content.
- Findings from studies investigating the mechanisms underlying this PV contraction have been controversial, possibly as experimental conditions were inadequately controlled.
- We examined the mechanisms underlying the PV contraction evoked by 4 days of exposure to hypobaric hypoxia (HH) in 11 healthy lowlanders, while strictly controlling water intake, diet, temperature and physical activity.
- Exposure to HH‐induced an ∼10% PV contraction that was accompanied by a reduction in total circulating protein mass, whereas diuretic fluid loss and total body water remained unchanged.
- Our data support an oncotically driven fluid redistribution from the intra‐ to the extravascular space, rather than fluid loss, as the mechanism underlying HH‐induced PV contraction.

**Abstract:**

Extended hypoxic exposure reduces plasma volume (PV). The mechanisms underlying this effect are controversial, possibly as previous studies have been confounded by inconsistent experimental conditions. Here, we investigated the effect of hypobaric hypoxia (HH) on PV in a cross‐over study that strictly controlled for diet, water intake, physical activity and temperature. Eleven males completed two 4‐day sojourns in a hypobaric chamber, one in normoxia (NX) and one in HH equivalent to 3500 m altitude. PV, urine output, volume‐regulating hormones and plasma protein concentration were determined daily. Total body water (TBW) was determined at the end of both sojourns by deuterium dilution. Although PV was 8.1 ± 5.8% lower in HH than in NX after 24 h and remained ∼10% lower thereafter (all *P* < 0.002), no differences were detected in TBW (*P* = 0.17) or in 24 h urine volumes (all *P* > 0.23). Plasma renin activity and circulating aldosterone were suppressed in HH during the first half of the sojourn (all *P *< 0.05) but thereafter similar to NX, whereas no differences were detected for copeptin between sojourns (all *P *> 0.05). Markers for atrial natriuretic peptide were higher in HH than NX after 30 min (*P* = 0.001) but lower during the last 2 days (*P *< 0.001). While plasma protein concentration was similar between sojourns, total circulating protein mass (TCP) was reduced in HH at the same time points as PV (all *P* < 0.03). Despite transient hormonal changes favouring increased diuresis, HH did not enhance urine output. Instead, the maintained TBW and reduced TCP support an oncotically driven fluid redistribution into the extravascular compartment as the mechanism underlying PV contraction.

## Key points

- Acclimatization to hypoxia leads to a reduction in plasma volume (PV) that restores arterial O_2_ content.
- Findings from studies investigating the mechanisms underlying this PV contraction have been controversial, possibly as experimental conditions were inadequately controlled.
- We examined the mechanisms underlying the PV contraction evoked by 4 days of exposure to hypobaric hypoxia (HH) in 11 healthy lowlanders, while strictly controlling water intake, diet, temperature and physical activity.
- Exposure to HH‐induced an ∼10% PV contraction that was accompanied by a reduction in total circulating protein mass, whereas diuretic fluid loss and total body water remained unchanged.
- Our data support an oncotically driven fluid redistribution from the intra‐ to the extravascular space, rather than fluid loss, as the mechanism underlying HH‐induced PV contraction.

## Introduction

During acute hypoxic exposure, an increase in cardiac output is required for the preservation of systemic O_2_ delivery in the face of reduced arterial oxygen content (CaO_2_) (Vogel & Harris, 1967). However, as exposure extends, CaO_2_ is restored by a progressive reduction in plasma volume (PV) and the resulting increase in arterial haemoglobin concentration ([Hb]) (Bärtsch & Saltin, 2008). Despite decades of research, the mechanisms underlying this PV contraction remain controversial.

The most widespread explanation is that PV decreases as a consequence of a reduction in total body water (TBW) caused by an increased diuresis commonly referred to as ‘altitude diuresis’ (Honig, 1989). While some studies have indeed observed a loss of TBW (Jain *et al*. 1980; Singh *et al*. 1986; Westerterp *et al*. 2000) or increased urine flow in extended hypobaric hypoxia (HH) (Zaccaria *et al*. 1998; Haditsch *et al*. 2015), others detected no effect on TBW (Sawka *et al*. 1996) or on diuresis (Bärtsch *et al*. 1988; Robach *et al*. 2002). Altitude diuresis has been attributed to suppression of the renin–angiotensin–aldosterone axis and vasopressin (Zaccaria *et al*. 1998; Loeppky *et al*. 2005) and/or an increase in atrial natriuretic peptide (ANP) (du Souich *et al*. 1987). Nevertheless, also the studies that investigated the effect of extended HH on these hormones have reported conflicting results, as recently reviewed (Siebenmann *et al*. 2017 *b*).

An alternative explanation is that hypoxia‐induced PV contraction does not reflect a loss of TBW, but a fluid shift from the intra‐ to the extravascular compartment driven by a decrease in total circulating protein mass (TCP) (Sawka *et al*. 1996). This is supported by studies reporting a HH‐induced PV contraction in the face of unchanged TBW (Westerterp *et al*. 1996) and/or a decrease in TCP (Westergaard *et al*. 1970; Young *et al*. 2019), although the latter is not a universal finding either (Siebenmann *et al*. 2015).

The conflicting results of past research may not only reflect the complexity and individual variability in the PV response to hypoxia, but also the difficulty of maintaining well‐controlled conditions during extended HH exposure. Studies were usually conducted at high altitude, where subjects were exposed to changes in water consumption, diet, physical activity and temperature; all factors known to independently affect PV (Sawka *et al*. 2000).

The aim of the present study was therefore to investigate the isolated effect of HH on PV by eliminating such confounding factors. In a cross‐over design, 11 male lowlanders were exposed to NX and HH equivalent to 3500 m altitude for 4 days each. Water intake, diet, physical activity, temperature and sleep were meticulously matched between sojourns. PV, TBW, urine output, volume‐regulating hormones and TCP were repeatedly measured and compared between sojourns. To avoid effects of diurnal variations, measurements were performed at the same time of day in both sojourns. Based on the results of our recent study (Siebenmann *et al*. 2015), we hypothesised that: (1) HH‐induced PV reduction reflects a loss of TBW due to increased diuresis; (2) the diuretic effects of HH are mediated by changes in volume‐regulating hormones; and (3) TCP is preserved in HH.

## Materials and methods

The study was approved by the ethics committee of the Bolzano Hospital, Italy (No 70–2019) and conducted in agreement with the *Declaration of Helsinki* (except registration in a database).

### Subjects

Eleven healthy, non‐smoking, male lowlanders (25 ± 4 years, 181 ± 8 cm, 72 ± 12 kg, body mass index: 22 ± 3 kg m^−2^) gave written informed consent to participate in the study. All subjects lived close to sea level and none of them had a high altitude ancestry or a history of acute mountain sickness (AMS) or other high altitude‐related illnesses. Subjects were of low to moderate physical activity and did not engage in regular endurance exercise. To avoid interference from previous hypoxic exposure, subjects refrained from visiting altitudes >2000 m in the month preceding and throughout the study.

### Protocol

After a medical examination, subjects completed two 4‐day sojourns in a 12 m × 6 m × 5 m (L × W × H) hypobaric chamber (terraXcube, EURAC Research, Bolzano, Italy, 262 m). One sojourn took place in NX under unmodified barometric pressure (741 ± 4 mmHg). During the other sojourn (HH), barometric pressure was reduced to 493 ± 0 mmHg, simulating an altitude of 3500 m. Six subjects started with the NX and five with the HH sojourn. The sojourns were separated by a 4‐week washout period.

Subjects reported to the laboratory on the evening before the sojourns and spent the night in our facilities. Both sojourns started at 06:00 the next morning, when, for the HH sojourn, the chamber was decompressed at a rate of 0.16 mmHg s^−1^ (corresponding to an ascent of 2 m s^−1^). After 4 days and nights in the chamber, both sojourns ended with final measurements on the morning of the fifth day. Throughout the sojourns, the mean temperatures were 22.7 ± 0.7°C and 22.0 ± 0.1°C and the relative humidity 26 ± 4% and 21 ± 2% in NX and HH, respectively.

Four days prior to and throughout both sojourns, the subjects followed the same standardised diet consisting of three main meals, three snacks and 3 l of water that was consumed in six 0.5 l portions at predefined times. The daily caloric intake was 2380 kcal (51% carbohydrate, 13% protein, 36% fat) and Na^+^ and K^+^ intakes were 110 and 97 mmol day^−1^, respectively. Alcohol was not allowed, and coffee consumption was limited to one cup per day.

Habitual physical activity was evaluated with a step‐counter over 4 days prior to the first sojourn. Based on this, individual daily step goals were defined, which the subjects were instructed to reproduce by walking on a treadmill while in the chamber. Subjects were free to split up their steps throughout the day as they wished. No other physical exercise was performed during the sojourns. During the daytime, subjects could move freely within the chamber or walk on the treadmill, whereas from 23:00 to 07:00 they were confined to bed and lights turned off.

### Measurements

**Figure 1:**
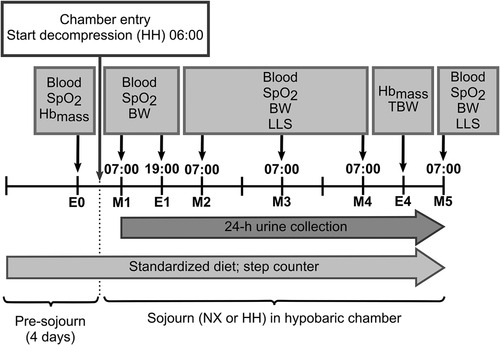
Schematic illustration of measurements performed during the normoxia (NX) and hypobaric hypoxia (HH) sojourn E0, evening before entering the hypobaric chamber; M1–M5, first to fifth morning of the sojourns; E1 and E4, first and fourth evening of the sojourns; blood, collection of a venous blood sample; SpO_2_, estimation of peripheral oxyhaemoglobin saturation by pulse oxymetry; Hb_mass_, determination of haemoglobin mass by carbon monoxide rebreathing; BW, body weight measurement; LLS, assessment of acute mountain sickness symptoms with the Lake Louise scoring system; TBW, assessment of total body water by deuterium dilution.

Venous blood samples were collected on the evening preceding the sojourns (E0) and then every morning (M1–M5) and on the first evening (E1) of the sojourns. Morning blood samples were collected at 07:00 while the subjects remained in bed, and evening samples were collected at 19:00 after 30 min of supine rest. Note that the M1 sample in the HH sojourn was collected 30 min after reaching the target pressure of 493 mmHg. During each sojourn, total blood withdrawal did not exceed 100 ml. At the same time points as blood samples were collected, peripheral oxyhaemoglobin saturation (SpO_2_) was estimated by pulse oxymetry (Nonin 150 WristOx2, US) and CaO_2_ was approximated as (1.34 × SpO_2_ × [Hb])/100, i.e. neglecting the small amount of O_2_ dissolved in plasma (Pittman, 2011). Total haemoglobin mass (Hb_mass_) was measured on E0 as well as on the last evening (E4) of both sojourns by carbon monoxide (CO) rebreathing for determination of intravascular volumes (see below). Body weight was measured every morning (M1–M5) of the sojourns after the first voiding. The incidence of AMS was evaluated with the Lake Louise AMS Scoring system (LLS) on M2–M5. The LLS is a self‐reported questionnaire consisting of five questions on typical AMS symptoms including headache, gastrointestinal symptoms, fatigue/weakness, dizziness/light‐headedness and sleep (Roach *et al*. 1993). Urine was collected continuously throughout both sojourns and TBW was measured over the last night by deuterium dilution. A schematic overview over the measurement time points is provided in Fig. 1.

### Blood analyses

Blood for haematocrit (Hct) and [Hb] analyses was collected into heparinised syringes. [Hb] and Hct were determined by blood gas analysis (ABL90 FLEX, Radiometer, Copenhagen, Denmark) and by the micro method (4 min at 13680 g), respectively.

Blood for plasma analyses was collected into anticoagulant‐covered vacutainers, separated by centrifugation (10 min at 1600 *g* and 4°C) and stored at −80°C for quantification of circulating hormones or at 4°C for protein and electrolyte analyses. Electrolyte concentrations ([K^+^], [Na^+^] and [Cl^−^]) were determined by an ion‐selective electrode (cobas, Roche Diagnostics). Creatinine ([Cr]) and plasma protein concentration (PPC) were measured by colorimetric assays (CREJ2, Roche Diagnostics and TP2 cobas, Roche Diagnostics, respectively). TCP was calculated as PPC × PV.

Plasma renin activity and aldosterone concentration were measured at steady state by radioimmunoassay as specified previously (Nussberger *et al*. 1984, 1987). Plasma concentrations of mid‐regional proANP (MR‐proANP), a stable marker for ANP, and of the vasopressin surrogate marker copeptin were performed on a Kryptor Plus automated platform (Thermo‐Fisher); the analytical validation of this method has been reported previously (Alehagen *et al*. 2011; Hunter *et al*. 2011). All plasma analyses were performed by blinded investigators.

### Haemoglobin mass and intravascular volumes

Hb_mass_ was measured by CO rebreathing as recently described (Siebenmann *et al*. 2017 *a*). Briefly, after 20 min of supine rest (including venous cannulation), subjects were connected to a mouthpiece, through which they breathed pure O_2_ from a Douglas bag for 4 min. Thereafter, they were switched via a sliding valve to an O_2_‐filled circuit consisting of a 6 l rebreathing bag and a CO_2_ scrubber. After collection of 2 ml of blood, a dose of ∼1.5 ml of CO per kg body weight was injected into the rebreathing circuit. A second blood sample was collected after 10 min of rebreathing and the volume of unabsorbed CO in the circuit was determined (Siebenmann *et al*. 2017 *a*). Blood samples were analysed in quadruplicate for the carboxyhaemoglobin fraction (%HbCO) by a blood gas analyser (ABL90 FLEX) and Hb_mass_ was determined from the volume of absorbed CO and the resulting change in %HbCO. Red blood cell volume (RBCV), PV and blood volume (BV) were derived for E0, M1–M5 and E1 by integration of Hb_mass_ and the Hct and [Hb] values determined at the respective time points with the following equations: (1) RBCV = Hb_mass_ × Hct/[Hb]; (2) BV = RBCV/Hct; (3) PV = BV ‐ RBCV (Siebenmann *et al*. 2017 *a*). Note that all intravascular volumes are entered in litres, Hct as an absolute fraction and Hb_mass_ and [Hb] in grams and grams/litre, respectively.

We used Hb_mass_ from the first CO rebreathing to derive intravascular volumes on E0, M1 and E1 and Hb_mass_ from the second rebreathing for intravascular volumes on M4 and M5. For M2 and M3, the average Hb_mass_ of the two CO rebreathings was used.

### Total body water

A stock solution containing 111.24 g of deuterium oxide (D_2_O, 99.88%, Cambridge Isotope Laboratories, Inc., MA, USA) and 1900 g of mineral water was prepared. In the evening, after voiding and before going to bed, a baseline saliva sample was collected. Subsequently, ∼80 g of the stock solution was ingested (the exact weight was recorded for each subject). The cups were then rinsed with 100 ml of tap water, which was also ingested by the subjects to ensure that all deuterium had been consumed. A second saliva sample was collected the next morning (10 h later). Subjects refrained from eating or drinking between saliva samples. To avoid an increase in daily water intake, subjects drank 200 ml less on day 4. Isotope enrichment relative to standard mean ocean water was determined in quadruplicate by isotope‐ratio mass spectrometry (Gasbench II – Conflo IV – Delta V advantage, Thermo scientific, Bremen, Germany). TBW was calculated as the deuterium dilution space divided by 1.04 to correct for non‐aqueous exchange using the formula provided by Schoeller *et al*. (1980).

### Urine analyses

Every 24 h, urine volumes were determined and a 6 ml sample of the 24 h urine was stored at 4°C for analyses of protein concentration, [Cr], [K^+^] [Na^+^] and [Cl^−^] by blinded investigators using the same methods as for plasma analyses.

### Renal filtration, reabsorption and excretion

Glomerular filtration rate (GFR) was calculated for each day based on Cr clearance as GFR = (([Cr]_urine_ × urine flow rate) / [Cr]_plasma_), where [Cr]_plasma_ is the average of the Cr concentration in plasma samples collected at the beginning and end of the 24 h urine collection and urine flow rate is the urine output (in ml) per minute derived from the 24 h urine volume. Daily excretion of Na^+^, Cl^−^ and K^+^ was calculated by multiplying the respective urine concentration with the 24 h urine volume. Tubular reabsorption of solutes was calculated as (GFR × average plasma solute concentration) ‐ solute excretion, and H_2_O reabsorption was calculated as GFR ‐ urine flow.

### Statistical analyses

Paired *t* tests were used to assess potential pre‐exposure differences between NX and HH on E0 (Table 1) and to compare the daily number of steps walked, Hb_mass_ and TBW between the two sojourns. The remaining data were analysed by mixed model for repeated measurements (MMRM) with fixed factors of altitude (NX *vs*. HH), order‐of‐sojourns (NX–HH, *N* = 6 or HH–NX, *N* = 5) and time‐point‐in‐chamber, and with random effect of subject. To isolate the effect of HH from any other effects of the chamber confinement, controlled diet, etc., pairwise comparisons, i.e. comparing the same time point during the NX sojourn with the corresponding one in HH, were performed as contrasts within the MMRM model and adjusted for multiplicity by Sidak's method. The study was not designed for examining changes over time. To preserve statistical power, changes over time within one chamber sojourn were not evaluated. Furthermore, as E0 measurements were exclusively performed to ensure that the pre‐exposure status of the subjects was comparable despite the 4‐week washout between the sojourns, these values were not included in the MMRM analyses or in the figures.

**Table 1:** Results collected on the evening before entering the chamber (E0) for both sojourns

	NX	HH	*P* value
	E0	E0	Paired *t* test
PV (ml)	3249 ± 609	3335 ± 543	0.067
Hb_mass_ (g)	815.2 ± 130.7	811.0 ± 129.1	0.429
BV (ml)	5681 ± 967	5770 ± 917	0.102
SpO_2_ (%)	98.0 ± 0.6	97.8 ± 0.8	0.441
CaO_2_ (ml dl^−1^)	18.9 ± 0.8	18.4 ± 0.7	0.008^**^
[Hb] (g dl^−1^)	14.4 ± 0.6	14.1 ± 0.5	0.005^*^
Hct (%)	43.0 ± 1.8	42.2 ± 0.8	0.083
PPC (g dl^−1^)	7.2 ± 0.2	7.1 ± 0.3	0.039^*^
TCP (g)	235.0 ± 45.4	235.8 ± 42.57	0.818
Renin activity (ng ml^−1^ h^−1^)	0.40 ± 0.25	0.38 ± 0.27	0.922
Aldosterone (pg ml^−1^)	83.5 ± 49.55	81.7 ± 39.26	0.891
Copeptin (pmol l^−1^)	3.67 ± 0.96	4.58 ± 2.00	0.044^*^
MR‐proANP (pmol l^−1^)	49.0 ± 24.5	46.7 ± 29.1	0.567
Plasma [Na^+^] (mmol l^−1^)	139 ± 1	139 ± 2	0.378
Plasma [Cl^−^] (mmol l^−1^)	101 ± 2	101 ± 1	0.714
Plasma [K^+^] (mmol l^−1^)	3.8 ± 0.2	3.9 ± 1.5	0.671

Abbreviations: PV, plasma volume; Hb_mass_, haemoglobin mass; BV, blood volume; SpO_2_, peripheral oxyhaemoglobin saturation; CaO_2_, arterial oxygen content; [Hb], venous haemoglobin concentration; Hct, haematocrit; MR‐proANP, mid‐regional pro‐atrial natriuretic peptide; PPC, plasma protein concentration; TCP, total circulating protein; NX, normoxia; HH, hypobaric hypoxia; E0, evening before entering the chamber. *N* = 11. Values are presented as means ± SD; ^*^ *P* < 0.05; ^**^ *P* < 0.01 HH *vs*. NX in paired *t* test.

A small number of measurements were missing; however, rather than imputing missing data, MMRM analysis better controls type I error and minimises bias. Data are presented as means ± SD and a *P* value <0.05 was considered statistically significant. The analysis was performed using SAS 9.4 (SAS Institute Inc., Cary, USA).

## Results

Values measured on E0 are presented in Table 1. While most variables were similar before the two sojourns, copeptin concentration was higher and [Hb], CaO_2_ and PPC were slightly, but significantly, lower before the HH sojourn. One subject presented with very high plasma renin activity (3.1 ng ml^−1^ h^−1^) and aldosterone (499 pg ml^−1^) and copeptin (381 pmol l^−1^) concentration on E0 before NX, which may have reflected a stress response (Gideon *et al*. 2020) since the subject was scared of the initial blood sampling. These values were considered outliers and excluded from analysis.

All subjects completed the NX and HH sojourn and none developed symptoms of AMS (LLS <3 at all times). The prescribed diet and water intake were closely adhered to and daily steps were almost identical between NX and HH (6812 ± 51 *vs*. 6840 ± 93 steps; *P* = 0.622). Due to difficulties with blood sampling, all measurements involving blood analyses are missing on M1 in HH for one subject. On day 2 in NX, one subject forgot to collect urine so that his urine volume, GFR, and electrolyte excretion and reabsorption could not be determined. Throughout both sojourns, six body weight measurements were missing in NX and two in HH.

On M1, SpO_2_ was reduced to 84.8 ± 2.6% in HH (*vs*. 98.1 ± 0.3% in NX, *P* < 0.001) and although it progressively increased to 90.0 ± 2.6% until M5, it remained significantly lower than in NX (*P* < 0.001 for all time points). CaO_2_ was reduced to 17.0 ± 1.1 ml dl^−1^ in HH on M1 (*vs*. 19.4 ± 0.9 ml dl^−1^ in NX, *P* < 0.001) and remained lower than in NX until M3 (*P* < 0.001 for E1 and M2, *P* = 0.028 for M3). On M4 and M5 in HH, CaO_2_ in was similar to NX (18.8 ± 0.7 *vs*. 19.4 ± 0.7 ml dl^−1^, *P* = 0.329 and 18.5 ± 0.9 *vs*. 19.2 ± 0.7 ml dl^−1^, *P* = 0.133, respectively) due to the increases in SpO_2_ and [Hb] (see below).

### Effects of hypobaric hypoxia on intravascular volumes

**Figure 2:**
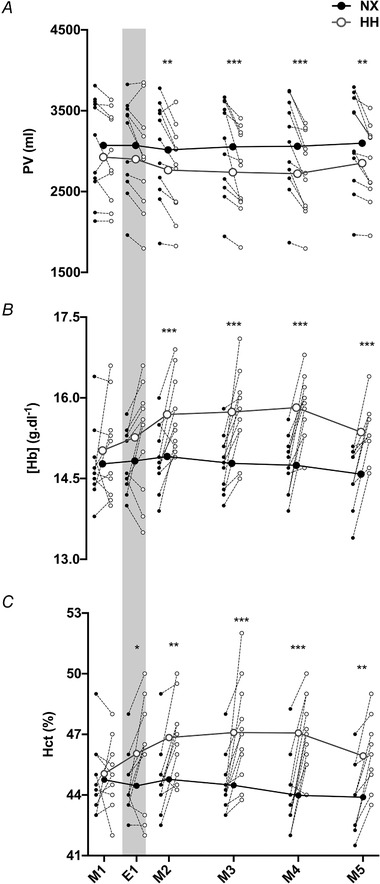
Effects of hypoxia on plasma volume Changes in plasma volume (PV) (*A*), venous haemoglobin concentration [Hb] (*B*) and haematocrit (Hct) (*C*) during a 4 day sojourn in normoxia (NX) and hypobaric hypoxia (HH). M1–M5, first to fifth morning of the sojourns; E1, first evening of the sojourns. Big symbols represent means, small symbols represent individual data;*N* = 11;^*^ *P* < 0.05;^**^ *P* < 0.01;^***^ *P* < 0.001 for time‐point comparisons between HH and NX by mixed model for repeated measurement (MMRM) analyses. Note that the grey shaded areas mark measurements performed in the evening whereas all other measurements were taken in the morning.

No differences were detected for Hb_mass_ between NX and HH on E0 (Table 1) or E4 (800.6 ± 129.2 g *vs*. 809.2 ± 127.3 g, *P* = 0.130). PV was similar between NX and HH on M1 (*P* = 0.746) and E1 (*P* = 0.059) (Fig. 2 *A*). On M2, PV was 251 ± 196 ml lower in HH than in NX (*P* = 0.001) and remained 250–350 ml lower for the rest of the sojourn (*P* < 0.001 for M3 and M4; *P* = 0.002 for M5). BV was similar between sojourns on E0 (Table 1) and M1 (NX: 5539 ± 988, HH: 5310 ± 921 ml, *P* = 0.395) but lower during the HH sojourn at the same time points as PV (all *P* < 0.008). While [Hb] was similar between sojourns on M1 and E1 (*P* = 0.678 and 0.140, respectively), it was higher in HH than in NX from M2 to M5 (all *P* < 0.001) (Fig. 2 *B*). Hct was not different in HH on M1 (*P* = 0.999) but thereafter higher than in NX for all time points (E1: *P* = 0.028; rest: *P* ≤ 0.002) (Fig. 2 *C*).

### Mechanisms of plasma volume contraction

**Figure 3:**
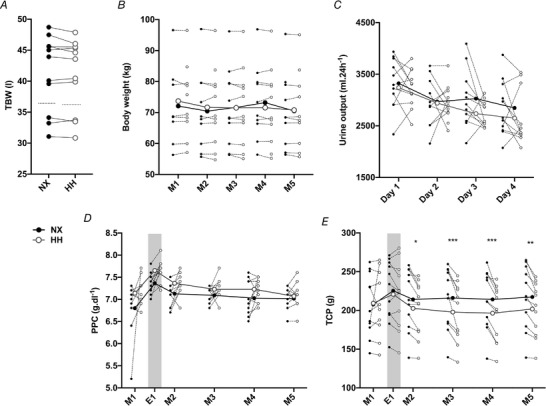
Mechanisms of plasma volume contraction *A*, individual values for total body water (TBW) assessed overnight between the fourth evening and the fifth morning in normoxia (NX) and hypobaric hypoxia (HH). Dotted lines represent the group means. Means were compared with paired*t*test.*B*, changes in body weight.*C*, daily urine output during the sojourns.*D*, plasma protein concentration (PPC).*E*, total circulating plasma protein (TCP) throughout the 4 day sojourns. M1–M5, first to fifth morning of the sojourns; E1, first evening of the sojourns (grey shade). Big symbols represent means, small symbols represent individual data;*N* = 11;^*^ *P* < 0.05;^**^ *P* < 0.01;^***^ *P* < 0.001 for time‐point comparisons between HH and NX by mixed model for repeated measurement (MMRM) analyses.

No difference was detected between NX and HH for TBW (41.3 ± 6.1 l *vs*. 41.0 ± 5.9 l, *P* = 0.171) (Fig. 3 *A*) or for body weight (*P* > 0.964 for all days) (Fig. 3 *B*). Furthermore, 24 h urine volume was at no point different between NX and HH (*P* > 0.230 for all days) (Fig. 3 *C*). While PPC was similar in NX and HH (all *P* > 0.070) (Fig. 3 *D*) TCP was 11.5 ± 10.1 g lower in HH on M2 (*P* = 0.029) and remained reduced until the end of the sojourn with a maximal difference of 18.3 ± 13.4 g on M3 (M2–M5: *P* < 0.002; M1–E1: *P* > 0.737) (Fig. 3 *E*). Urine protein concentration was low throughout both sojourns, remaining below the detection limit (4 mg dl^−1^) in almost half of the measurements.

### Volume‐regulating hormones

**Figure 4:**
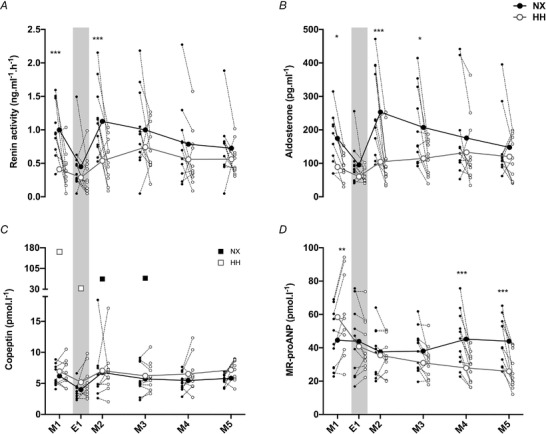
Volume‐regulating hormones Changes in renin (*A*), aldosterone (*B*), copeptin (*C*) and mid‐regional pro‐atrial natriuretic peptide (MR‐proANP) (*D*) concentrations during the normoxia (NX) and hypobaric hypoxia (HH) sojourn. For graphing purposes, values that exceeded the normal range by more than 200% were excluded from the mean and SD in (*C*). The values are plotted individually as black (NX) and white (HH) squares. Big symbols represent means, small symbols represent individual data;*N* = 11;^*^ *P* < 0.05;^**^ *P* < 0.01;^***^ *P* < 0.001 for time‐point comparisons between HH and NX by mixed model for repeated measurement (MMRM) analyses. Grey shaded areas mark evening measurements.

Plasma renin activity was lower in HH than NX on M1 and M2 (both *P* < 0.001) but similar at all other time points (all *P* > 0.326) (Fig. 4 *A*). Plasma aldosterone concentration was lower in HH on M1 (*P* = 0.049), M2 (*P* < 0.001) and M3 (*P* = 0.021) but not on E1, M4 and M5 (all *P* ≥ 0.675) (Fig. 4 *B*). No differences were observed for plasma copeptin concentrations between the two sojourns (*P* = 0.110 for M1 and *P* > 0.970 for all other time points) (Fig. 4 *C*). MR‐proANP concentration was higher in HH than in NX on M1 (*P* = 0.001) and lower on M4 (*P* < 0.001) and M5 (*P* < 0.001), whereas no significant differences were observed on E1, M2 and M3 (all *P* > 0.173) (Fig. 4 *D*). Note that on E1 all hormones were similar between sojourns (all *P* > 0.815).

### Renal water and electrolyte handling

**Figure 5:**
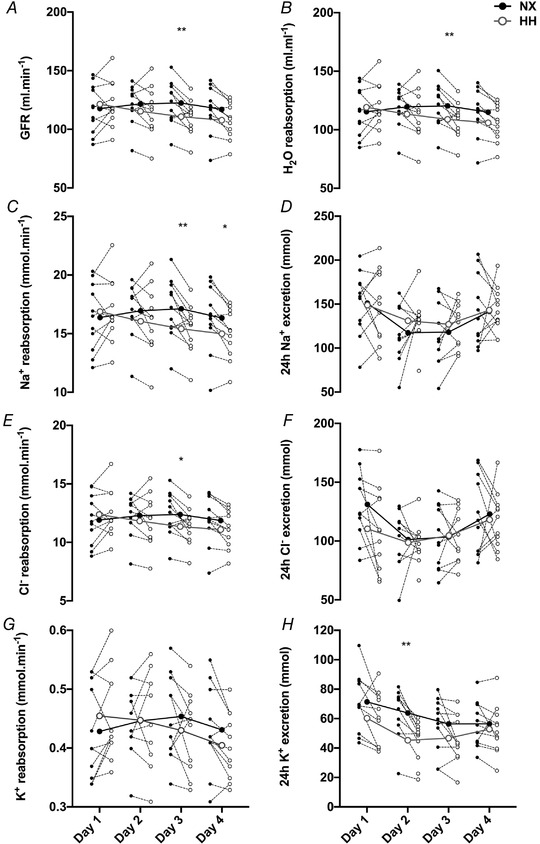
Renal water and electrolyte handling Changes in glomerular filtration rate (GFR) (*A*) and tubular H_2_O reabsorption (*B*) during days 1–4 in normoxia (NX) and hypoxia (HH). Changes in electrolyte reabsorption (*C, E*and*G*) and excretion (*D, F*and*H*) throughout the sojourns. Big symbols represent means, small symbols represent individual data;*N* = 11;^*^ *P* < 0.05;^**^ *P* < 0.01 for time‐point comparisons between HH and NX by mixed model for repeated measurement (MMRM) analyses.

GFR (Fig. 5 *A*) and tubular H_2_O reabsorption (Fig. 5 *B*) were lower in HH than in NX on day 3 (*P* = 0.008 and *P* = 0.009, respectively), whereas no significant differences were detected on days 1–2 (all *P* > 0.503) or on day 4 (*P* = 0.056 and *P* = 0.059, respectively). Na^+^ reabsorption (Fig. 5 *C*) was lower in HH on days 3 and 4 (*P* = 0.006 and *P* = 0.043, respectively; all other *P* > 0.486), whereas Na^+^ excretion (Fig. 5 *D*) did not differ between the two sojourns (all *P* > 0.440). Reabsorption of Cl^−^ was lower in HH on day 3 (*P* = 0.035; all other *P* > 0.176) (Fig. 5 *E*) while Cl^−^ excretion was similar to NX (all *P* > 0.163; Fig. 5 *F*). No difference was observed for K^+^ reabsorption between NX and HH (all *P* > 0.317) (Fig. 5 *G*) but K^+^ excretion was lower in HH on day 2 (*P* = 0.002; day 1: *P* = 0.066; days 3–4: *P* > 0.133) (Fig. 5 *H*). Plasma electrolyte concentrations are provided in Table 2. Plasma [Na^+^] was similar between NX and HH at all time points, whereas [Cl^−^] was higher in HH on M2, M3 and M5 and [K^+^] was higher in HH on M3 (Table 2).

**Table 2:** Plasma electrolyte concentrations during two 4 day sojourns in normoxia (NX) and hypobaric hypoxia (HH)

[Na^+^] (mmol l^−1^)						
	M1	E1	M2	M3	M4	M5
NX	140 ± 1	139 ± 2	140 ± 1	140 ± 1	141 ± 1	140 ± 1
HH	140 ± 1	140 ± 2	140 ± 1	140 ± 2	140 ± 2	140 ± 1
*P* values	1.000	0.999	1.000	0.999	0.337	0.999

[Cl^−^] (mmol l^−1^)						
	M1	E1	M2	M3	M4	M5
NX	102 ± 2	100 ± 2	101 ± 2	101 ± 2	102 ± 2	102 ± 1
HH	102 ± 2	101 ± 2	103 ± 2	103 ± 2	103 ± 2	104 ± 1
*P* values	0.999	0.766	0.018^*^	0.006^**^	0.192	0.001^***^

[K^+^] (mmol l^−1^)						
	M1	E1	M2	M3	M4	M5
NX	4.1 ± 0.3	3.9 ± 0.2	4.1 ± 0.3	4.0 ± 0.2	4.0 ± 0.2	4.1 ± 0.3
HH	4.1 ± 0.2	3.8 ± 0.2	4.1 ± 0.2	4.3 ±0.3	4.1 ± 0.2	4.1 ± 0.3
*P* values	0.933	0.408	1.000	0.001^***^	0.724	0.946

Abbreviations: M1–M5, first to fifth morning in the chamber; E1, first evening in the chamber; NX, normoxia; HH, hypobaric hypoxia. Values represent means ± SD; *N* = 11; ^*^ *P* < 0.05; ^**^ *P* < 0.01; ^***^ *P* < 0.001 for time‐point comparisons between HH and NX by mixed model for repeated measurement (MMRM) analyses.

## Discussion

We investigated the mechanisms underlying hypoxia‐induced PV contraction in a hypobaric chamber, while meticulously controlling for factors that typically confound field studies at high altitude. Four days of HH equivalent to 3500 m altitude reduced PV by ∼10%. Contrary to our hypotheses, and despite transient hormonal changes favouring diuretic water loss, this PV reduction was not associated with increased diuresis and a consequent loss of TBW. Instead, TCP decreased during HH, supporting an oncotically driven fluid redistribution from the intra‐ to the extravascular space.

PV contraction is an almost universal finding in healthy lowlanders exposed to HH equivalent to altitudes >2000 m (Siebenmann *et al*. 2017 *b*), although exceptions occur in individuals developing AMS (Hackett *et al*. 1982; Bärtsch *et al*. 1988; Loeppky *et al*. 2005) or when HH exposure is combined with strenuous exercise (Levine & Stray‐Gundersen, 1997; Gore *et al*. 1998). In the current study, PV decreased primarily during the first 24 h of HH, and changed little thereafter, which aligns with a recent model predicting the time course of PV changes at high altitude (Beidleman *et al*. 2017) and also the magnitude of PV reduction was in the same range (6–14%) as in previous studies at altitudes of ∼3500 m (Siggaard‐Andersen *et al*. 1968; Jung *et al*. 1971; Singh *et al*. 1986; Siebenmann *et al*. 2015).

HH‐induced PV contraction is often attributed to a decrease in TBW (Jain *et al*. 1980; Singh *et al*. 1986; Westerterp *et al*. 2000) due to enhanced diuretic water loss (Zaccaria *et al*. 1998; Haditsch *et al*. 2015). Nevertheless, other studies have reported PV reduction in the face of an unchanged TBW (Westerterp *et al*. 1996; Sawka *et al*. 1996) and diuresis (Bärtsch *et al*. 1988; Robach *et al*. 2002) at altitude. The conflicting results of past research possibly reflect inadequate control of water intake, physical activity, temperature and diet. In the present study, where these confounding factors were strictly controlled for, TBW was not different between HH and NX. While HH exposure is often associated with a loss of body weight (Dünnwald *et al*. 2019), we have not observed any weight changes throughout the sojourns, which probably not only reflects the controlled diet and the low physical activity but also supports the finding that TBW remained stable. PV constitutes only ∼20% of extracellular fluid volume and is in unrestricted exchange with the extravascular compartment (Sawka *et al*. 2015). Accordingly, an extracellular fluid and thus TBW loss of ∼1.7 l would have been required for the 339 ml PV reduction observed on M4 in HH. In line with the unchanged TBW, we also observed no effect of HH on 24 h urine output. In contrast, two well‐controlled studies detected a marked increase in urine flow after 90 min (Hildebrandt *et al*. 2000) or 6 h (Swenson *et al*. 1995) of normobaric hypoxia (12% O_2_). A potential explanation is that normobaric hypoxia with 12% O_2_, which corresponds to an altitude of ∼4300 m, triggers an increase in diuresis that does not occur in the milder hypoxia used in the present study. Alternatively, acute hypoxia might induce increases in urine flow that are followed by compensatory reductions so that the 24 h urine volume remains unchanged. Indeed, Loeppky *et al*. have observed increased urine flow during the first 9 h in HH (in subjects not affected by AMS), whereas after 12 h, urine flow was lower than in normoxia (Loeppky *et al*. 2005). Nevertheless, even if such an early, transient increase in diuresis occurred in our study, its effect on PV must have been minor, since on E1 (i.e. after 12 h) PV was not yet significantly lower in HH than in NX.

In addition to diuresis, it has been suggested that enhanced natriuresis contributes to PV reduction in hypoxia (Swenson *et al*. 1995; Zaccaria *et al*. 1998), which was, however, not confirmed by the present and earlier studies (Hildebrandt *et al*. 2000; Loeppky *et al*. 2005).

The paradigm of altitude diuresis is also based on studies reporting suppression of the renin–angiotensin–aldosterone axis (Hogan *et al*. 1973; Zaccaria *et al*. 1998) and/or of vasopressin (Claybaugh *et al*. 1978; Loeppky *et al*. 2005), although none of these are consistent findings (Siebenmann *et al*. 2017 *b*). Due to its small size and short half‐life, vasopressin is technically difficult to quantify in plasma and we therefore measured copeptin, which is a stable and sensitive surrogate marker for vasopressin (Morgenthaler, 2010), and was unaffected by HH. However, we observed a transient reduction in plasma renin activity and aldosterone concentration. Of note, these reductions were only detected in the morning, but not in the evening. Renin and aldosterone follow diurnal variations, with lower values in the evening (Hurwitz *et al*. 2004), potentially leaving little margin for further suppression by hypoxia (Kurtz, 2012). The decreased aldosterone probably caused a reduction in sodium reabsorption that attenuated water reabsorption by the connecting tubule and the collecting duct (Fig. 5 *B–C*). The fact that these effects did not increase diuresis and natriuresis implies a reduced GFR and hence reduced filtered load of sodium and water. GFR was indeed lower in HH from day 2 to 4, although statistical significance was only reached on day 3 (Fig. 5 *A*). This reduction in GFR was likely a consequence of reduced renal plasma flow due to the HH‐induced haemoconcentration (Pichler *et al*. 2008). Another hormone that has been proposed to mediate hypoxia‐induced diuresis is ANP (Honig, 1989). However, the effect of hypoxia on ANP is controversial as increases have been observed during acute normobaric hypoxia (<60 min) (Kawashima *et al*. 1989; Vonmoos *et al*. 1990), and unchanged or reduced values after HH exposure lasting several days (Bärtsch *et al*. 1988; Zaccaria *et al*. 1998; Siebenmann *et al*. 2015). Collectively, these results suggested a biphasic response, which is confirmed by the current results, where an increase in MR‐proANP was observed after 30 min in HH and a reduction after 3 and 4 days, respectively. While the transient increase in ANP may contribute to the increase in urine flow observed during acute normobaric hypoxia (Swenson *et al*. 1995; Hildebrandt *et al*. 2000), it is apparently too short‐lived to exert an increase in 24 h urine output.

Apart from increased diuresis, water loss via sweat, transcutaneous evaporation, ventilation or faeces can reduce TBW (Sawka *et al*. 2015). In the present study, temperature and physical activity were matched between sojourns, making differences in sweat loss unlikely. As diet was controlled, and none of the subjects had gastrointestinal symptoms, a systematic difference in faecal water loss between sojourns seems improbable as well (Westerterp *et al*. 1996). However, respiratory and transcutaneous water loss could have been higher during HH due to lower humidity (Sawka *et al*. 2015) and increased pulmonary ventilation (Wagner *et al*. 1987). Nevertheless, as TBW was unchanged, such increases in respiratory and transcutaneous water loss must have been small, which is in line with investigations of fluid balance at altitude (Westerterp *et al*. 2000).

A reduction in PV occurs in the face of unchanged TBW if fluid is redistributed from the intra‐ to the extravascular space and this is an alternative, albeit less widespread, explanation for hypoxia‐induced PV contraction. Several studies have found HH to promote a loss of TCP (Westergaard *et al*. 1970; Sawka *et al*. 1996; Young *et al*. 2019) and the resulting reduction in oncotic pressure could drive a transvascular fluid shift and thus reduce PV. In line with this, PV reduction in HH occurred in parallel with a decrease in TCP in the present study. A loss of plasma protein can occur in hypoxia through multiple processes: (i) transvascular escape (Westergaard *et al*. 1970), (ii) increased degradation (Surks, 1966), or (iii) increased excretion in urine (Hansen *et al*. 1994). The latter did not, however, occur in the present study as urine protein concentrations were mostly below the detection limit (4 mg dl^−1^). Even when assuming a concentration of 4 mg dl^−1^ for these values, daily protein excretion was below 140 mg throughout both sojourns and hence cannot explain the 18 g loss of TCP during HH. In contrast to the present results, we have previously reported unchanged TCP during 4 weeks of exposure to 3454 m (Siebenmann *et al*. 2015). A potential explanation is that in the previous study the time points of blood sampling were not as closely controlled as in the current study. Figure 3 *E* suggests that TCP is higher in the evening than in the morning and such diurnal variations could have prevented the detection of the mild (∼5–8%) reduction in TCP observed in the present study. Furthermore, the uncontrolled diet in the previous study could have been associated with a higher protein intake that maintained TCP by accelerating albumin synthesis (Thalacker‐Mercer & Campbell, 2008).

Apart from reducing PV, hypoxic exposure also promotes Hb_mass_ expansion through accelerated erythropoiesis. However, as expected (Rasmussen *et al*. 2013; Siebenmann *et al*. 2015), 4 days in HH were insufficient for this effect to manifest. Despite this, CaO_2_ was restored to NX levels at the end of the HH sojourn, which is in line with earlier findings at 3454 m altitude (Siebenmann *et al*. 2015) and highlights the functional importance of PV contraction for blood O_2_ transport capacity.

There are methodological aspects to consider: a 4‐day chamber confinement with a controlled diet, physical activity and sleep/wake cycle represents a lifestyle change that, in itself, could affect PV and other variables measured. To isolate the effects of hypoxia from those of chamber confinement, a NX sojourn was performed and variables were compared at specific time points between the HH and NX sojourns. To preserve statistical power, changes occurring over time during a single sojourn, which would reflect the combined effect of HH and chamber confinement, were not evaluated. Similarly, the E0 measurements, which were exclusively performed to ensure that the pre‐exposure status of the subjects was comparable despite the 4‐week washout between the sojourns, should not be interpreted as baseline measurements. As these measurements were performed in the evening preceding the sojourns, any PV change occurring from E0 to M1 would not only reflect the effect of 30 min of HH, but also of the onset of chamber confinement as well as circadian variations (Schmidt *et al*. 2000). E0 values were therefore not included in the MMRM analyses or in the figures.

We chose a severity of HH that induces a clear PV contraction without posing too high a risk for AMS. The latter could not only have led to subject dropouts but also interfered with water homeostasis due to fluid retention (Loeppky *et al*. 2005) or fluid loss due to gastrointestinal symptoms (Westerterp *et al*. 1996). While previous studies performed at similar altitudes reported an AMS incidence of ∼30–40% (Honigman *et al*. 1995; Beidleman *et al*. 2013, 2018; Phillips *et al*. 2017), none of the subjects in the present study developed AMS. A part of the explanation for the discrepancy may be that a history of AMS or other high altitude‐related illnesses was an exclusion criterion. In addition, strenuous physical exercise, which may enhance the risk for AMS (Beidleman *et al*. 2013), was avoided during the sojourns. The controlled water intake also ensured that subjects remained well‐hydrated, which could have prevented AMS (Cumbo *et al*. 2002; Castellani *et al*. 2010). Finally, as AMS commonly strikes during the first night at altitude (Luks *et al*. 2017), the absence of AMS might have been related to the early ascent, which extended the acclimatization time preceding the first night.

The exposure duration of 4 days was selected as major changes in PV are known to take place within this period (Siebenmann *et al*. 2015, 2017 *b*; Beidleman *et al*. 2017). Accordingly, it seems unlikely that a different mechanism for PV reduction would have been recruited after a longer exposure.

Finally, we included only males as variations in PV (Cullinane *et al*. 1995), (anti‐)diuretic hormones (Jensen *et al*. 1989; Chidambaram *et al*. 2002) and PPC (Ferris *et al*. 1962) across the menstrual cycle would have complicated the replication of experimental conditions between NX and HH. Follow‐up studies evaluating whether the current results apply to women are hence warranted.

In conclusion, HH in the absence of confounding variables reduces PV by promoting oncotically driven fluid shifts from the intra‐ to the extravascular space. No indications of the increased urine output commonly referred to as ‘altitude diuresis’ were observed despite transient suppression of renin and aldosterone, and a short‐lived increase in circulating ANP. These findings advance our understanding of the normal PV response to HH and facilitate future investigations on the pathophysiology of fluid retention during AMS.

## Additional information

### Competing interests

The authors have no conflicts of interest to declare.

### Author contributions

The experiments were performed at the terraXcube at Eurac Research in Bolzano, Italy. C.S. and E.F. designed and planned the research. All listed authors acquired, analysed and interpreted the data. M.S. and C.S. drafted the article and all authors critically revised and approved the final version. All listed authors agree to be accountable for all aspects of the work and ensure that questions related to the accuracy or integrity of any part of the work are appropriately investigated and resolved. All persons designated as authors qualify for authorship, and all those who qualify for authorship are listed.

### Funding

This work was funded by The Swiss National Centre of Competence in Research (NCCR) Kidney Control of Homeostasis (Kidney.CH) (C.S. and E.F.).

## Supporting information


**Statistical Summary Document**

## Data Availability

The data that support the findings of this study are available from the corresponding author upon reasonable request.
